# Ultrasonic Degradation of Konjac Glucomannan and the Effect of Freezing Combined with Alkali Treatment on Their Rheological Profiles

**DOI:** 10.3390/molecules24101860

**Published:** 2019-05-14

**Authors:** Bo Zhu, Chen Xin, Jing Li, Bin Li

**Affiliations:** 1 College of Food Science and Technology, Huazhong Agricultural University, Wuhan 430070, China; zhubo283999192@163.com (B.Z.); xinchen951@163.com (C.X.); libinfood@mail.hzau.edu.cn (B.L.); 2 Key Laboratory of Environment Correlative Dietology, Huazhong Agricultural University, Ministry of Education, Wuhan 430070, China; 3 Training Base of Army Logistics, University of PLA, Xiangyang 441000, China

**Keywords:** konjac glucomannan, ultrasonic degradation, freezing treatment, rheological properties

## Abstract

The effect of freezing combined with alkali treatment on physicochemical property of konjac glucomannan (KGM) with different molecular weight was investigated in this work. The properties and structure of degraded KGM was characterized by means of intrinsic viscosity measurement, atomic force microscope (AFM) and Fourier transformation infrared (FT-IR). The results suggested that the intrinsic viscosity of KGM solution gradually decreased during the ultrasonic treatment. The AFM observation indicated that KGM with lower viscosity average molecular weight had smaller height and lateral diameter of molecules. The main repeating units of the KGM chain could not be destroyed no matter how long the KGM was sonicated. Rheometrical studies revealed that with increasing alkali concentration from 0% to 0.36%, both viscosities and shear stress of deacetylated konjac glucomannan (Da-KGM) system were increased and moduli G′ were substantially higher in either freezing or unfreezing samples. Da-KGM system performed a solid-like behavior (G′ > G′′) along the frequency range after freezing treatment. With increasing sonication time, both viscosity and shear stress of unfreezing samples were decreased while had an inverse effect for freezing treated samples. The modulus G′ and G′′ declined for unfreezing samples but rise significantly for freezing treated samples with increase of sonication time.

## 1. Introduction

**Figure 1:**
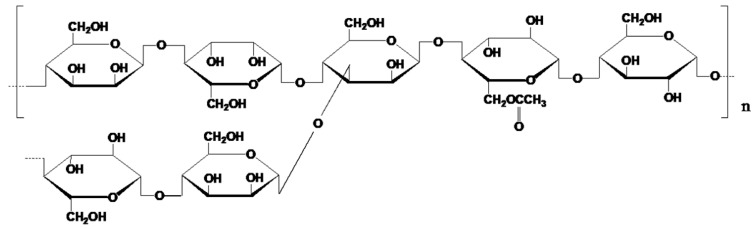
The chemical structure of konjac glucomannan.

Konjac glucomannan (KGM) is a kind of natural neutral polysaccharide which extracted from konjac tuber. It consists of β-1, 4-linked °d-mannose and °d-glucose, with some short branches. The usual ratio of mannose and glucose in KGM lies between molar ratios of 1:1.5 and 1:1.6 with a high degree of substitution (5∼10%) of the OH groups by acetyl groups (Figure 1) [1]. In the presence of alkali, KGM will release the acetyl and form a heat stable gel that is the basis of many traditional oriental foods. With an increasing degree of deacetylation, water solubility of KGM decreased, and hydrophobic interaction was strengthened whilst hydrogen bonding was weakened [2]. The health benefits of KGM as dietary fiber have been widely reported, such as relieving constipation, controlling body weight and regulating blood glucose [3]. Hence KGM is often added to foods for its health effects [4]. The prebiotic and antioxidative potential of degraded konjac glucomannan were also reported. The study of Connolly and Lovegrove suggested that the unique properties of degraded KGM make it valuable as a prebiotic [5]. Wang et al. found the fermentation of hydrolysed KGM produced antioxidative capacity by increasing the radical-scavenging ability and eliminating lipid peroxide formation [6].

In our previous studies, ultrasonic treatment was found to be an effective approach for KGM degradation [7]. Ultrasound is a kind of mechanical wave with a frequency of above 20 Hz. The depolymerization process occurs by the cavitation effect involving two possible mechanisms: mechanical degradation of the polymer from collapsed cavitation bubble and chemical degradation on account of the chemical reaction between the high-energy and polymer molecules, for example, the hydroxyl radicals produced from cavitation phenomenon [8]. Because ultrasonic treatment involves many pyrolytic and radical induced degradation processes, a series of degradation products were detected, and many mechanisms were proposed. It has been widely investigated on ultrasonic degradation of polysaccharides. More and more researchers have focused on the physico-chemical properties of ultrasound-treated polysaccharides [9,10]. Sonication of high-amylose maize starch caused a significant reduction in intrinsic viscosity but showed similar ^1^H NMR spectra [11]. High intensity ultrasound could also effectively decrease the intrinsic viscosity of the sodium alginate solution; besides, amplitude and time of the sonication had a direct effect on the viscosity degradation, but the sonication temperature had an inverse effect [12].

KGM with different molecular weights exhibited distinct characteristics. Lower molecular weight samples of KGM showed slippage in both the storage modulus (G′) and loss modulus (G′′) because a rapid gelation process with syneresis and the disentanglement of molecular chains adsorbed on the surface of parallel plates from those located in the bulk [13]. The critical gelation temperature of KGM increased with the increasing molecular weight [14]. Both intrinsic viscosity and sedimentation coefficient decreased with KGM molecular weight decreased [15]. Generally, the molecular structure influences the rheological and gel properties of KGM [16]. The properties of KGM have been confirmed to influence the rheological, textural and water holding properties of the food system in which it is used [17,18].

According to our previous studies, freezing treatment had a great impact on rheology of Da-KGM. This phenomenon has also been observed by other researchers [19,20]. In this work, the effect of ultrasonic time on structure and properties of KGM and rheology of freezing treated Da-KGM with ultrasonic degradation were investigated. It is expected to develop ultrasonic degradation combined with freezing as a means to regulate the rheological properties of KGM so as to further expand its application in healthy food.

## 2. Results and Discussion

### 2.1. Effects of Ultrasonic Time on the Intrinsic Viscosity and Viscosity Average Molecular Weight of KGM Solutions

The intrinsic viscosity [ɳ], correlation (R^2^) and molecular weight ($M_{\eta }$) (*M* *_ɳ_*) of KGM solutions, which correspond to no-sonication, 5 min, 15 min, 30 min, or 60 min sonication were shown in Table 1. The result showed a good fit to the Huggins equation as it showed higher correlation (R^2^). Therefore, the estimation of the molecular weight by Huggins equation seemed to provide more reliable results. The viscosity average molecular weight of the sample treated for 0, 5, 15, 30 and 60 min was decreased from the initial 1.02 × 10^6^ to 6.36 × 10^5^, 3.93 × 10^5^, 2.10 × 10^5^ and 1.22 × 10^5^, respectively. It indicated that a relatively long-time sonication treatment lead to the degradation of polysaccharides. After sonication, since the molecular chains became shorter, the influence of molecular interaction on intrinsic viscosity became limited and less important. Such phenomenon was also observed in xanthan gum [21], locust bean gum [22] and polysaccharides from *Porphyra yezoensis* [23].

### 2.2. Effects of Ultrasonic Time on the Molecular Morphology of KGM

**Figure 2:**
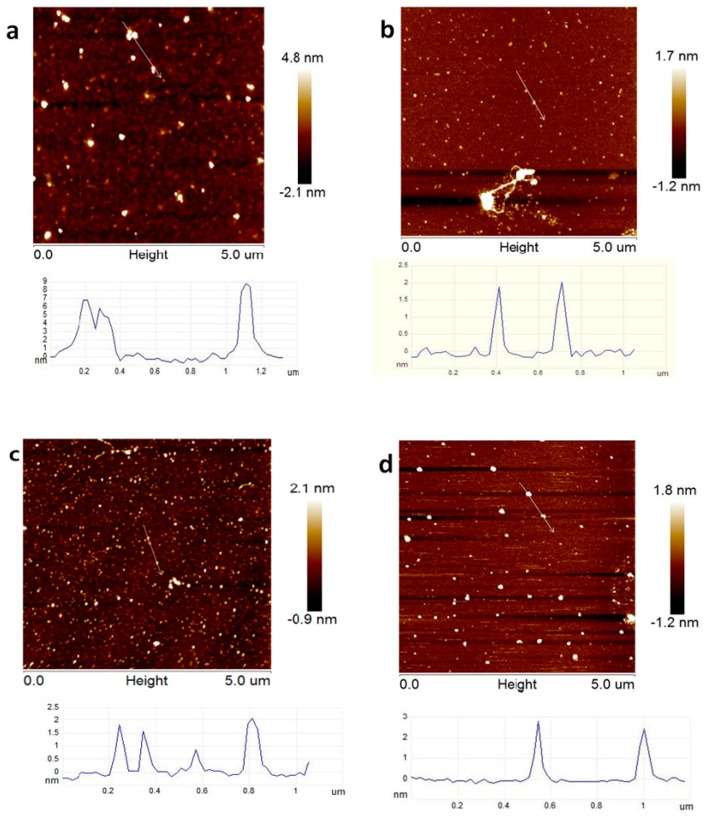
AFM image of KGM treated with different ultrasonic time (image size = 5 × 5 µm); (**a**) non–sonication, (**b**) 15 min, (**c**) 30 min, (**d**) 60 min.

The molecular morphology of different ultrasonic time was obtained by AFM (Figure 2). It was observed that both the height and lateral diameter of non-sonication KGM molecules (Figure 2a) were significant bigger than the ultrasonically treated ones [24]. We assumed that during ultrasound treatment, degradation of KGM in aqueous solutions was happened and led to the decrease of the height and lateral diameter of KGM molecules. While there were no significant differences between the molecules with different ultrasonic time. Additionally, after one-hour sonication (Figure 2d), Intermolecular associations could be observed in dilute solution.

### 2.3. FT-IR Analysis

**Figure 3:**
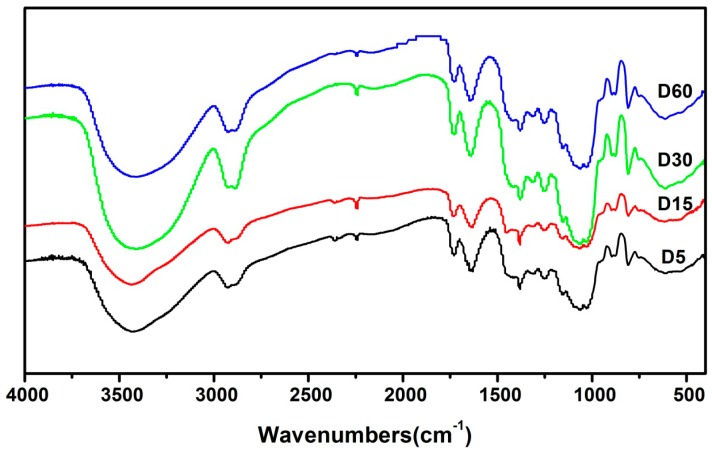
FT-IR spectra of KGM treated with different ultrasonic time (D5, D15, D30 and D60 was assigned to KGM prepared by ultrasonication for 5, 15, 30 and 60 min, respectively).

FT-IR is of importance in studying molecular structure. Figure 3 showed the FT-IR spectra of KGM with different ultrasonic time, the wavenumber ranged of 4000∼400 cm^−1^. The band absorbance was matched with the vibrational modes of the chemical bonds by lots of researchers. The absorption band in the peak at 3400 cm^−1^ was assigned to the stretching of –OH groups. The bands in the region of 2926.6 cm^−1^ and 1380 cm^−1^ was assigned to the stretching and bending vibration of C-H. The band in the region of 895 cm^−1^ and 809.5 cm^−1^ was assigned to mannose existence. Absorbance at 1735cm^−1^ was assigned to the stretching of carbonyl groups, which could detect the presence of acetyl groups. These results were in agreement with the data reported by Jin et al. [25]. Moreover, the similarity of the FT-IR spectra of KGM treated by different ultrasonic times indicated the main repeating units of the KGM chain were not destroyed no matter how long the KGM was sonicated.

### 2.4. Rheological Characterization

#### 2.4.1. Effect of Alkali Concentration on Rheological Profiles

**Figure 4:**
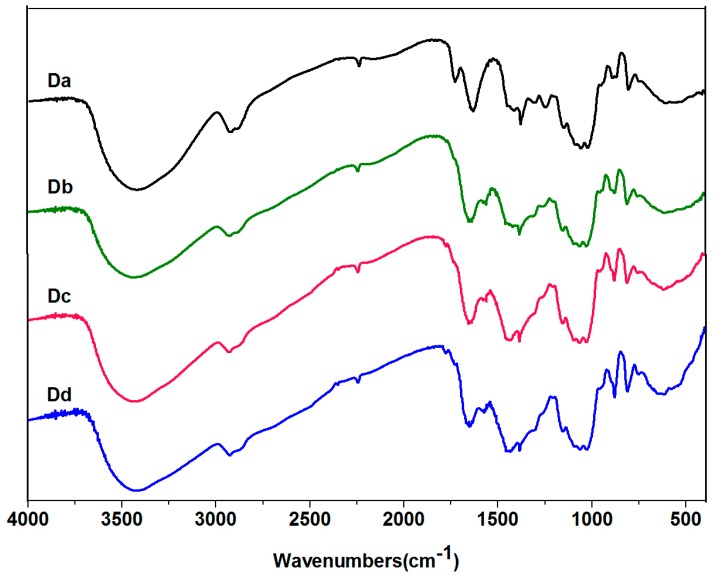
FT-IR spectra of KGM treated under the different alkali concentration (Da, Db, Dc and Dd was assigned to KGM treated under the different alkali concentration of 0%, 0.12%, 0.24%, and 0.36%, respectively).

**Figure 5:**
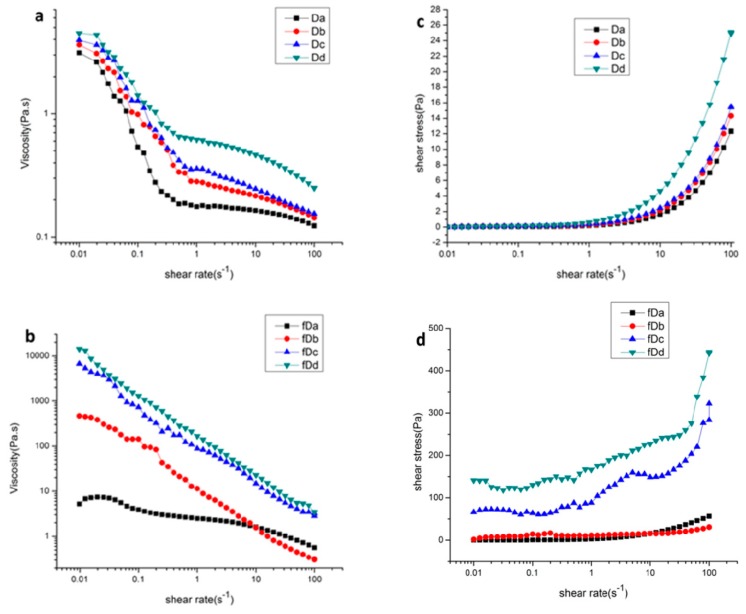
The steady state flow curves of KGM sols with different deacetylation degree before and after freezing dealt.

The effect of alkali concentration on the flowing and frequency sweep curves of Da-KGM before and after freezing treatment was first studied. The fractions treated under the alkali concentration for 0%, 0.12%, 0.24%, and 0.36% were named Da, Db, Dc, and Dd, respectively and freezing samples treated with different alkali concentration were named fDa, fDb, fDc, and fDd, respectively. Figure 4 showed the FT-IR spectra of KGM treated with different amounts of alkali. The most obvious change was that except for Da, the peak of other samples disappeared at 1735 cm^−1^, where the peak was the stretching vibration peak of the carbonyl group on the acetyl group, indicating that the addition of alkali could effectively remove the acetyl group of konjac glucomannan. As can be seen from Figure 5a,b, the shear rate sweeps (0.01∼100 s^−1^) of the viscosity showed that the viscosities of all samples decreased when the shear rate increased revealed a shear thinning behavior of the samples, indicating they belonged to the non-Newtonian fluids and owned the pseudoplastic characteristics. As found in the previous report of Xu et al. [26], that type of shear-thinning behavior was usual for random coil polysaccharides [27]. Briefly, at lower shear rate, the rate of disentangle of Da-KGM molecule became greater than the rate of re-entanglement and the effect could be amplified with increasing shear rate. As a consequence, the intermolecular resistance to flow reduced and a lower apparent viscosity appeared.

With alkali concentration increased from 0% to 0.36%, the viscosities of Da-KGM system were increased no matter unfreezing and freezing treated samples. More than at least hundreds fold increase in viscosity was observed in freezing treated Da-KGM system (fDb, fDc & fDd) compared to unfreezing Da-KGM (Db, Dc & Dd).

Figure 5c,d showed a plot of shear stress versus shear rate for the unfreezing and freezing treated samples, respectively. The shear stress of unfreezing samples kept invariant at low shear rate and increased rapidly at high shear rate. The shear stress of unfreezing and freezing treated samples was increased with increasing alkali concentration from 0% to 0.36%.

The power law б = Kɣ^n^ was widely used to evaluate the equality of KGM, the parameters K and n were displayed in Table 2. Index n is a measure of the pseudoplastic flow and K is a measure of the liquid viscosity. Polymer dispersions also exhibit shear thinning or thickening behavior which results in n < 1. The extent of shear thinning or thickening depends on some intrinsic and extrinsic parameters including polymer properties such as size, shape and concentration of macromolecules in solution, presence of ions, solvent type and temperature. With the increase of alkali concentration, the index n decreased, and index K increased. The index n of unfreezing Da-KGM was higher than that of freezing treated Da-KGM. And index K of freezing treated Da-KGM was much higher than unfreezing samples. So it was clear that both freezing treatment and relatively high alkali concentration could make it behaves in pseudoplastic manner. These results were in agreement to the previous study [28]. Because KGM consists of 5–10% of the –OH groups by acetyl substituted residues, which inhibited the formation of intramolecular hydrogen bonding in the dilute solutions; this keeps the molecule in an extended form, which in turn increases the degree of pseudoplasticity [29].

**Figure 6:**
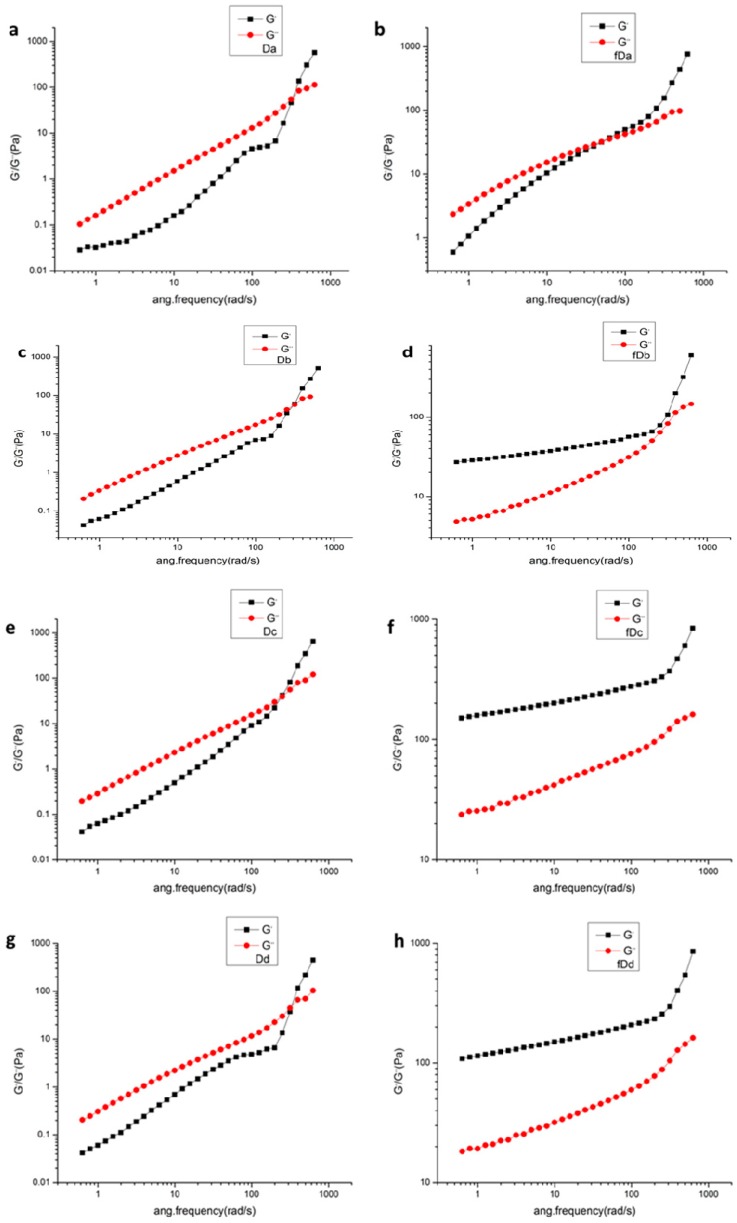
Frequency dependence of G′ and G′′ of KGM sols with different alkali addition before freezing (**a**,**c**,**e**,**g**) and after freezing (**b**,**d**,**f**,**g**) dealt.

The effect of alkali concentration on the frequency sweep curves of Da-KGM before and after freezing treatment was shown in Figure 6. Frequency sweeps were performed over a range of 0.5∼100 Hz at strain of 1% to determine the dependence of G′ and G′′ as functions of frequency. For unfreezing Da-KGM, the modulus G′′ was higher than the modulus G′ at low frequencies and they both increased with frequency increasing, performing the dominant liquid characteristic. While at higher frequencies, the behavior approached to solid-like materials. It can be explained that at lower frequencies, molecular chains disentangled during a long period of oscillation; yet at higher frequencies, KGM molecules can’t disentangle during a short period of oscillation and form a temporary network structure. The cross over point of unfreezing samples were barely changed. The sample of fDa (native KGM) showed similar dynamic viscoelastic properties to unfreezing samples because no deacetylation happened in the absence of alkali. But fDb, fDc, fDd (deacetylated KGM) system performed a solid-like behavior (G′ > G′′) at all frequencies, which promoted the formation of larger and more frequent junctions for denser and more elastic networks. All levels of additional alkali increased G′ and G′′ significantly of freezing treated system compared to unfreezing samples. Interestingly, the variation of G′ with different Na_2_CO_3_ concentrations was significant. The moduli G′ were substantially higher with increased of Na_2_CO_3_ concentrations for unfreezing and freezing treated samples. So predominantly elasticity behavior was exhibited in the presence of Na_2_CO_3_.

#### 2.4.2. Effect of Sonication Time on Rheological Profiles

**Figure 7:**
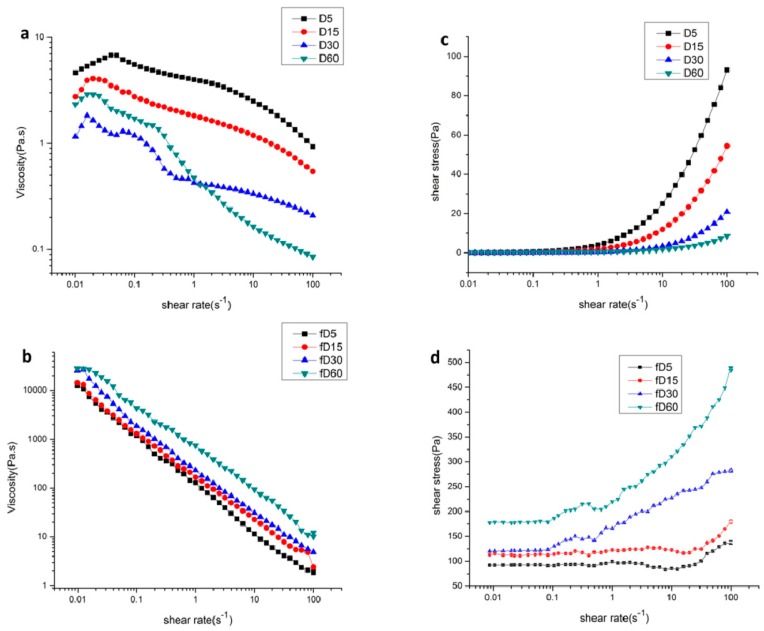
The steady state flow curves of 1% KGM sols with different sonication time before and after freezing dealt.

**Figure 8:**
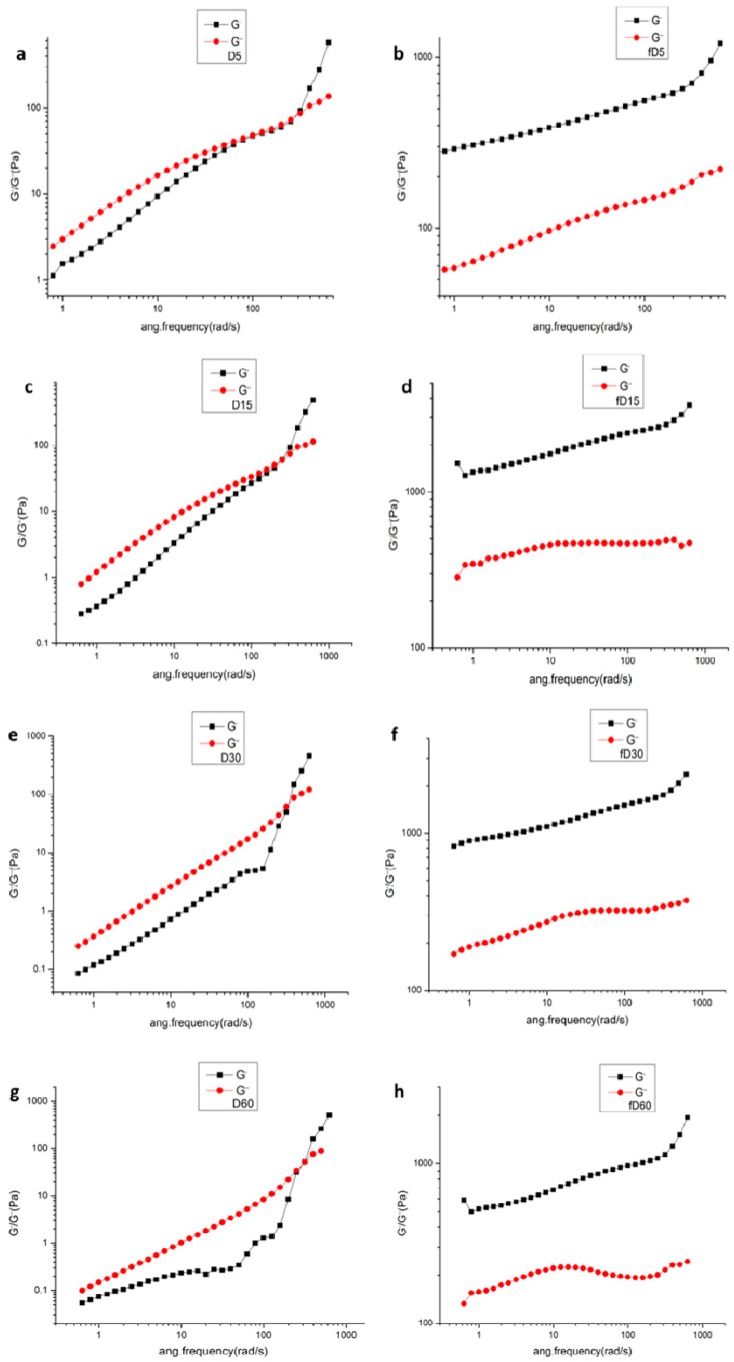
Frequency dependence of G′ and G′′ of 1% KGM sols with different sonacition time before freezing (**a**,**c**,**e**,**g**) and after freezing (**b**,**d**,**f**,**h**) dealt.

The effects of sonication time on the flowing and frequency sweep curves of Da-KGM before and after freezing treatment were shown in Figure 7 and Figure 8. The fractions treated under the ultrasonic conditions for 5, 15, 30, and 60 min were named D5, D15, D30, and D60, respectively and freezing treated samples with different ultrasonic time were named fD5, fD15, fD30, and fD60, respectively.

The influence of sonication time on steady shear properties of Da-KGM before and after freezing treatment was shown in Figure 7. The increase of shear rates led to shear thinning behavior. Viscosities of unfreezing samples decreased with increasing ultrasonic time. The viscosity of a polysaccharide solution depends on many factors such as molecular mass, stiffness and charge of the molecule. Therefore, the distinct viscosity might due to the differences in the molecular mass of the four samples where the molecular mass was decreased with ultrasonic time increased. When the polymer with higher molecular weight exists in solutions, the more interactions can be established by entanglements and hydrogen, electrostatic and hydrophobic bonds [30]. However, the viscosity of all samples increased dramatically after freezing, indicating that freezing enhanced the interaction between Da-KGM molecules. 

Figure 7c,d showed a plot of shear stress versus shear rate for the unfreezing and freezing treated samples, respectively. Shear stress at all rates decreased with increasing sonication time for unfreezing samples indicating a reduction in dispersion viscosity, while shear stress was increased with increasing sonication time for freezing treated samples. 

The drastic influence of sonication time can also be seen from fits of the flow curve to the power law model б = Kɣ^n^. Table 3 showed a plot of the power law indexes K and n of the Da–KGM system for unfreezing and freezing treated samples. D30 were observed to exhibit the highest index n in unfreezing samples, which indicated a shift towards a more Newtonian behavior. An ideal Newtonian fluid has a power law index of *n* = 1. The index n of unfreezing Da-KGM was higher than that of freezing treated Da-KGM. And index K of freezing treated Da-KGM was far more than higher than unfreezing samples. Therefore, it was clear that freezing treated Da-KGM system behaved pseudoplastic. It was believed that freezing treated Da-KGM process better quality with increasing ultrasonic time because n was smaller, and K became larger. Therefore, it was clear that sonication had greatly improved the liquid properties.

Figure 8 showed the results of frequency sweeps with different ultrasonic time before and after freezing dealt. The untreated Da-KGM sols exhibited a typical concentrated solution bonding phenomenon [31], the modulus G′′ was higher than the modulus G′ at low frequencies and lower than modulus Gʹ at high frequencies. With increase of ultrasonic time, the crossover between viscous and elastic behavior of the unfreezing Da-KGM was unchangeable but modulus G′ and G′′ were gradually decreased. It may be attributed to the degradation of KGM molecules. Similar phenomenon was observed in KGM–xanthan mixtures with different molecular weight of KGM [14]. In all cases, the storage modulus G′ was greater than the loss modulus G′′ for freezing treated samples which was irrespective of sonication time. This phenomenon clearly showed the mechanical spectra typical of polysaccharide gels: G′ was always much greater than G′′ and is nearly independent of the frequency over a wide frequency range [32]. In addition, the modulus G′ and G′′ of freezing treated Da-KGM increased significantly with the increase of ultrasonic treatment time, and reached maximum when the treated time was 15 min, then went down with the further increase of ultrasonic treated time. These indicated that the gel strength of Da-KGM gel formed after freezing treatment was related to the molecular weight of KGM.

## 3. Material and Methods

### 3.1. Materials

Konjac glucomannan was purchased from konson konjac Gum Co., Ltd (Hubei, China). Sodium chloride (NaCl), sodium carbonate (Na_2_CO_3_) and potassium bromide (KBr) were purchased from Sinopharm Chemical Reagent Co., Ltd (Shanghai, China).

### 3.2. Ultrasonic Treatment of Konjac Glucomannan

The 1% (*w*/*w*) KGM sol were prepared by suspending KGM in distiller water and stirring with a mechanical stirrer at medium speed for 1 h. Ultrasound treatment was carried out using probe ultrasonic processor (Fisher Scientific, FB705, Pittsburgh, USA). The probe ultrasonic processor had a maximum power of 700 W, operated at a frequency of 20 kHz. The sol (200 mL) was placed in a glass beaker, which was immersed into ice bath to avoid overheating, and performed in a way of treating 5 s with 2 s cooling intervals. The ultrasonic probe was submerged into the sol 2 cm from the top surface of the sol. The KGM sols were treated with ultrasound at 80% amplitudes for different times (0 min, 5 min, 15 min, 30 min and 60 min).

### 3.3. Intrinsic Viscosity of KGM Solutions

A sample for intrinsic viscosity measurement was prepared by dispersing KGM in 0.2 mol/L NaCl, stirred overnight and then filtered through a Watman No. 1 filter paper to remove insoluble components. The ubbelohde viscometer (0.5∼0.6 mm) was immersed in a precision water bath maintained at 25.0 ± 0.1 °C. Serial dilution was performed in situ and three readings were taken for each dilution after equilibration and averaged.

The sample viscosity (η) was converted to specific viscosity (η_sp_) and relative viscosity (η_rel_) using Equations (1) and (2), respectively:

(1) $$\eta_{sp}=\eta_{rel}-1=\frac{\eta -\eta_{s}}{\eta_{s}}$$

(2) $$\eta_{rel}=\frac{\eta }{\eta_{s}}$$

where *η_s_* is the viscosity of the solvent.

The intrinsic viscosity [η] is often obtained from the extrapolation of lnη_rel_/c or η_sp_/c to infinite dilution according to the Huggins and Kraemer empirical expressions as follows:

Huggins equation [33]:

(3) $$\frac{\eta_{sp}}{C}=[\eta ]+k_{1}[\eta ]{}^{2}C$$

Kraemer equation [34]:

(4) $$\frac{In\eta_{rel}}{C}=[\eta ]+k_{2}[\eta ]{}^{2}C$$

The viscosity average molecular weight (*M_ɳ_*) can be estimated by using the following Mark-Houwink equation [35]:

(5) $$[\eta ]=k\times M_{\eta }^{\alpha }$$

where *k* and *α* are 5.96 × 10^−2^ and 0.73, respectively.

### 3.4. Atomic Force Microscopy (AFM) Measurement

KGM solution (1 × 10^−3^ mg/mL) were observed by a multimode-8 scan probe microscope (Bruker, Herzogenrath, Germany). The konjac glucomannon aqueous solutions (8 µL) were deposited onto a freshly cleaved mica substrate and allowed to dry. Contact mode in air was employed in this study under 1 Hz scan rate and 256 × 256 pixel image resolution. The experiment was carried out at room temperature (20 °C) and 35% relative humidity.

### 3.5. Fourier Transformation Infrared (FT-IR) Analysis

Samples were mixed with KBr (1:50) and pressed into KBr pellets prior to FT-IR analysis. FT-IR spectra were collected by a 470 FT-IR (Nicolet Nexus, Waltham, USA) at the absorbance mode in the frequency range of 4000∼400 cm^−1^, with a resolution of 4 cm^−1^.

### 3.6. Preparation of Deacetylated Konjac Glucomannan (Da-KGM) Sols

In order to investigate the effect of freezing treatment on rheological properties of KGM treated with different concentrations of alkali, different amount of Na_2_CO_3_ was added into KGM (ultrasonic treated for 0 min) sol with stirring for 1 h and standing for 3 h. The final concentration of KGM was 1 wt%. The final concentrations of Na_2_CO_3_ solution were 0 wt%, 0.12 wt%, 0.24 wt% and 0.36 wt%, respectively.

In order to investigate the effect of sonication time on rheological properties of freezing treated Da-KGM, Na_2_CO_3_ solution was added into the KGM (ultrasonic treated for 5 min, 15 min, 30 min and 60 min, respectively) sols with stirring for 1 h and standing for 3 h to remove the acetyl groups attached to the saccharide units. The final concentration of KGM and Na_2_CO_3_ solution were 1 wt% and 0.36 wt%, respectively.

### 3.7. Preparation of Freezing Treated Da-KGM Sols

Da-KGM sols were put in ice refrigerator (−20 °C) for 36 h and thawed at 30 °C.

### 3.8. Rheological Characterization

The viscosity of the samples was determined by rheometer (AR2000, TA Instruments, Newcastle, UK) with a parallelplate (diameter 40 mm, 1 mm gap). Flow curves with increasing shear rate (0.01∼100 s^−1^) were measured at a constant temperature of 25 °C. The viscosity versus shear rate data was fit by the power law mode. Oscillatory measurements were used to determine the storage modulus (G′) and loss modulus (G′′) of the samples. Frequency sweep (0.5∼100 Hz at 1% stain) was conducted from 0.5 to 100 Hz at 1% stain, which is within a linear viscoelastic region.

## 4. Conclusions

In this work, the effect of freezing treatment on konjac glucomannan with different molecular weight was investigated. After sonication for at most 60 min, the molecular weight of KGM was from initial 1.02 × 10^6^ to 1.22 × 10^5^ at last. The results of AFM indicated that KGM with lower molecular weight had smaller height and lateral diameter of molecules. The main repeated units of the KGM chain were not destroyed no matter how long the KGM was sonicated according to FT-IR analysis. The rheology study showed that the viscosities of KGM sols decreased when the shear rate increased revealed a shear thinning behavior of the samples. With increasing alkali concentration from 0% to 0.36%, both viscosities and shear stress of Da-KGM system were increased and moduli G′ were substantially higher no matter unfreezing and freezing treated samples. Da-KGM system performed a solid-like behavior (G′ > G″) at all frequencies after freezing treatment. With increasing sonication time, both viscosity and shear stress of unfreezing samples were decreased while had an inverse effect for freezing treated samples. It is obvious that with relative long sonication, the modulus G′ and G′′ decreased for unfreezing samples but increased significantly for freezing treated samples.

## Figures and Tables

**Table 1:** Effect of sonication time on intrinsic viscosity and molecular weight of KGM sols.

Sonication Time (min)	$$\frac{\eta_{sp}}{C}=[\eta ]+k_{1}[\eta ]{}^{2}C$$		$$\frac{In\eta_{rel}}{C}=[\eta ]+k_{2}[\eta ]{}^{2}C$$		Molecular Weight $\left(M_{\eta }\right)$
	[η](dl/g)	R^2^	[η](dl/g)	R^2^	
**0**	1456.01	0.977	1522.24	0.764	1.02 × 10^6^
**5**	1027.23	0.957	1043.67	0.642	6.36 × 10^5^
**15**	723.08	0.950	730.00	0.614	3.93 × 10^5^
**30**	458.35	0.988	470.00	0.968	2.10 × 10^5^
**60**	309.06	0.988	315.09	0.972	1.22 × 10^5^

**Table 2:** Parameters of KGM sols with different amount of alkali dealt before and after freezing treatment estimated by Power law equation.

No.	n	K(Pa·s)	No.	n	K(Pa·s)
Da	0.8613	0.2354	fDa	0.5757	4.128
Db	0.8146	0.3388	fDb	0.1528	12.52
Dc	0.8040	0.3802	fDc	0.1316	125.2
Dd	0.7278	0.8908	fDd	0.1091	177.2

**Table 3:** Parameters of the Da-KGM system treated for different sonication time estimated by Power law equation.

No.	n	K(Pa·s)	No.	n	K(Pa·s)
D5	0.5909	6.372	fD5	0.1686	131.1
D15	0.6629	2.629	fD15	0.1179	135.2
D30	0.7897	0.5558	fD30	0.1091	177.2
D60	0.7050	0.3577	fD60	0.9873	263.9
